# Whole-genome assembly and evolutionary analysis of the *Malus kansuensis* (Rosaceae) mitochondrion

**DOI:** 10.1080/23802359.2021.2005473

**Published:** 2021-11-29

**Authors:** Lingli Li, Xiting Gu, Jianwen Ma

**Affiliations:** College of Forestry, Northwest A&F University, Yangling, China

**Keywords:** *Malus kansuensis*, mitochondrial genome, phylogenetic analysis

## Abstract

*Malus kansuensis*, which belongs to the family Rosaceae, is an important apple rootstock resource in China. In the present study, the mitochondrial genome of *M. kansuensis* was sequenced and assembled by high-throughput sequencing (HTS). The genome was 385,436 bp in length, with an obvious (A + T) % bias over (G + C) %. The mitochondrial genome comprised 35 protein-coding genes, 21 tRNA genes, and 3 rRNA genes. The phylogenetic tree results showed that *M. kansuensis* is very close to *M. domestica* and *M. hupehensis.*

The *Malus kansuensis* (Batalin) C. K. Schneid. is a special Rosaceae family wild fruit tree resource in China (Wang et al. 2013). Because of its well-developed root system, dwarfing habits, high resistance to abiotic and biotic stress and early flowering, it is an important apple rootstock resource (Zhang et al. 2010). Plant mitochondrial genome data constitute a large number of unexplored and potentially rich sources of phylogenetic information (Rydin et al. 2017). Here, the mitochondrial genome of *M. kansuensis* was sequenced and assembled by high-throughput sequencing (HTS) technology, and its phylogenetic relationships in the genus *Malus* were also analyzed.

The leaves of *M. kansuensis* were collected from plant innovation teaching and experimental base at Northwest A&F University (Yangling City, Shannxi Province, China; 34.262678 N, 108.069433 E). The leaves of *M. kansuensis* specimens (WUK 0031609) were preserved in the herbarium of Northwest A&F University. Genomic DNA was extracted by a DNeasy Plant Mini Kit (Qiagen, USA) and sequenced on the Illumina HiSeq X platform (approximately 11.5 Gbp clean reads). The mitochondrial genome was assembled with MITObim software (Hahn et al. 2013). Genome annotation was performed in the online program GeSeq (Tillich et al. 2017) and compared with the *Malus* x *domestica* (NC_018554.1) mitochondrial genome. The graphical map of the genome was generated by the OGDRAW program (Lohse et al. 2013).

The *M. kansuensis* mitochondrial genome was a 385,436 bp circular DNA molecule. The mitochondrial base composition was A 26.6%, T 26.5%, G 21.8%, and C 22.2%, with an obvious (A + T) % bias over (G + C) %. Subsequently, the structure and organization of the genome was identified through the web-based tool Public MITOFY Analysis (http://dogma.ccbb.utexas.edu/mitofy/). In the mitochondrial genome, 59 functional genes were predicted, including 35 protein-coding genes, 21 tRNA genes and 3 rRNA genes. Most functional genes appeared to have one copy, and only 5 genes had two or more copies.

In addition, a phylogenetic analysis was performed using the protein-coding genes from the mitochondrial genomes of 8 species within Rosales (Figure 1). The *Manihot esculenta* mitochondrial genome was used as an outgroup. A phylogenetic tree was constructed by the maximum-likelihood method (1000 bootstraps) under the GTRGAMMAI substitution model in MEGA6 (Tamura et al. 2013). It showed that the *M. kansuensis* is very close to *M. domestica* and *M. hupehensis*. We hope that the results will further supplement the genomic information on mitochondrial of the Rosaceae family and facilitate the study of population genomic analysis and phylogenetic relationships for this species.

**Figure 1.**
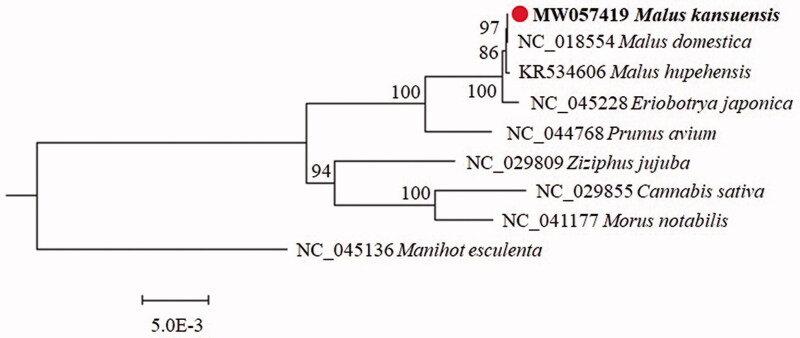
The phylogenetic tree inferred from 9 mitochondrial genomes. The *Malus kansuensis* complete mitochondrial genome obtained in this study is shown in bold. The number at each node is the bootstrap probability. The number in the species name is the GenBank accession number.

## Data Availability

The genome sequence data that support this study are openly available in GenBank of the NCBI at (https://www.ncbi.nlm.nih.gov/) under accession no. MW057419.2. The associated BioProject, SRA, and BioSample numbers are PRJNA765500, SRR16018185 and SAMN21561968, respectively. The specimen and data also available from the correspondence author Lingli Li, lill@nwafu.edu.cn.
